# The emergence of influenza B as a major respiratory pathogen in the absence of COVID-19 during the 2021–2022 flu season in China

**DOI:** 10.1186/s12985-023-02115-x

**Published:** 2023-08-24

**Authors:** De Chang, Mingui Lin, Ning Song, Zhantao Zhu, Jing Gao, Shujun Li, Hongmei Liu, DeZhi Liu, Yu Zhang, Wenkui Sun, Xuan Zhou, Bin Yang, Yongjun Li, Lili Wang, Zhiqing Xiao, Kailong Li, Lihua Xing, Lixin Xie, Lokesh Sharma

**Affiliations:** 1 College of Respiratory and Critical Care Medicine, Chinese PLA General Hospital, Beijing, 100083 China; 2 Department of Pulmonary and Critical Care Medicine, 7th Medical Center of Chinese, PLA General Hospital, 100007 Beijing, China; 3 Beijing Tsinghua Changgung Hospital, Tsinghua University School of Medicine, Beijing, 102218 China; 4 Department of Infectious Diseases, The Second Hospital of Hebei Medical University, Shijiazhuang, 050000 Hebei China; 5 Third Medical Center, Chinese PLA General Hospital, Beijing, 100039 China; 6 Department of Respiratory Intensive Care Unit, The First Affiliated Hospital of Zhengzhou University, Zhengzhou, 450052 Henan China; 7 The First Affiliated Hospital of Xinxiang Medical University, Weihui, 453199 Henan China; 8 Department of Respiratory and Critical Care Medicine, Henan Provincial People’s Hospital, Zhengzhou, 450003 Henan China; 9 Department of Pediatrics, Xinxiang Central Hospital, Xin Xiang, 453000 Henan China; 10 Department of Respiratory & Critical Care Medicine, The First Affiliated Hospital of Nanjing Medical University, Nanjing, 210029 Jiangsu China; 11 Department of the Second People’s Hospital of Hefei, Hefei Hospital Affiliated to Anhui Medical University, Anhui 230011 Hefei, China; 12 Vision Medicals Center for Infectious Diseases, Guangzhou, 510000 Guangdong China; 13 Department of Biochemistry and Biophysics, Peking University Health Science Center, Beijing, 100191 China; 14 Section of Pulmonary and Critical Care and Sleep Medicine, Yale University School of Medicine, New Haven, CT 06520-8057 USA

**Keywords:** Influenza B, COVID-19, Respiratory tract infection, Pneumonia

## Abstract

**Background:**

The emergence of COVID-19 and the implementation of preventive measures and behavioral changes have led to a significant decrease in the prevalence of other respiratory viruses. However, the manner in which seasonal viruses will reemerge in the absence of COVID-19-related restrictions remains unknown.

**Methods:**

Patients presenting with influenza-like illness in two hospitals in Beijing were subjected to testing for COVID-19, influenza A, and influenza B to determine the causative agent for viral infections. The prevalence of influenza B across China was confirmed using data from the Centers for Disease Control, China (China CDC). Clinical characteristics, laboratory findings, imaging results, and mortality data were collected for a cohort of 70 hospitalized patients with confirmed influenza B from 9 hospitals across China.

**Results:**

Starting from October 2021, a substantial increase in the number of patients visiting the designated fever clinics in Beijing was observed, with this trend continuing until January 2022. COVID-19 tests conducted on these patients yielded negative results, while the positivity rate for influenza rose from approximately 8% in October 2021 to over 40% by late January 2022. The cases started to decline after this peak. Data from China CDC confirmed that influenza B is a major pathogen during the season. Sequencing of the viral strain revealed the presence of the Victoria-like lineage of the influenza B strain, with minor variations from the Florida/39/2018 strain. Analysis of the hospitalized patients' characteristics indicated that severe cases were relatively more prevalent among younger individuals, with an average age of 40.9 ± 24.1 years. Among the seven patients who succumbed to influenza, the average age was 30 ± 30.1 years. These patients exhibited secondary infections involving either bacterial or fungal pathogens and displayed elevated levels of cell death markers (such as LDH) and coagulation pathway markers (D-dimer).

**Conclusion:**

Influenza B represents a significant infection threat and can lead to substantial morbidity and mortality, particularly among young patients. To mitigate morbidity and mortality rates, it is imperative to implement appropriate vaccination and other preventive strategies.

## Introduction

Influenza infections have been a leading cause of death worldwide, with an estimated annual mortality rate of approximately half a million people [5, 11]. Furthermore, the economic burden associated with influenza is substantial, estimated at $87.1 billion in 2007 [9]. However, the landscape of respiratory viral infections has undergone a significant transformation due to the emergence of COVID-19 and the implementation of precautionary measures. In the past two years, common respiratory infections such as influenza have seen a drastic reduction globally, attributed to widespread lockdowns, mask usage, and potentially viral interference caused by COVID-19 [4, 8, 16]. Understanding the future trajectory of influenza infections is of critical importance to mitigate their impact on public health.

Notably, China, particularly outside of Wuhan, managed to maintain relatively low COVID-19 case numbers through various measures including travel restrictions, contact tracing, mass testing, and extended quarantine [1]. As a result, areas such as Beijing, where limited or no COVID-19 cases were detected, returned to a state of relative "normalcy." It remains unclear how these measures have affected the spread of common respiratory viruses like influenza and whether they have allowed for the resurgence of previously prevalent strains or altered the prevalence of influenza strains. Additionally, the impact of respiratory infections on individuals who may have developed enhanced susceptibility due to the prolonged absence of common respiratory viruses remains unclear.

In this study, we aimed to identify the causative agent of respiratory infections among individuals presenting with respiratory symptoms at fever clinics. We confirmed this through analysis of influenza data obtained from the China CDC. Subsequently, we collected clinical and biological parameters from hospitalized individuals with influenza B infection to compare the characteristics of those who experienced severe disease or died as a result of influenza B infection.

## Methods

### Design

This study was a retrospective observational multicenter study that incorporated data from multiple sources. The data sources included: (i) Two fever clinics located in two different districts of Beijing, China. These clinics were utilized to identify the causative agent of viral infection (ii) Data from the Centers for Disease Control, China (China CDC) were accessed to confirm the prevalence of influenza B across China. (iii) Clinical data from hospitalized patients with influenza B infection were collected from nine hospitals located in five different provinces in China. The study design, including these data sources, is summarized in Table 1.

**Table 1:** Study design indicating different sources of data and samples analyzed in the study

Purpose	Source of data	Samples collected	Data analyzed	Figure/Table
Find the causative agent of influenza-like disease in Beijing	Two hospitals in Beijing	Nasal swabs of 8 patients for viral sequencing	Prevalence of influenza B/Sequence of influenza B	Figures 1A &B, 2A and B
Validate the presence of influenza B	China CDC	None	Influenza B cases in China	Figure 3
To determine the clinical characteristics of patients hospitalized with influenza B	Nine hospitals across 5 provinces of China	None	Clinical data	Tables 2, 3 and 4

Our primary objective was to determine the causative agent of a significant ongoing viral infection in Beijing, specifically in the absence of COVID-19. To achieve this, we conducted the study at two hospitals: the Chinese PLA General Hospital (Hospital 1) and Beijing Tsinghua Changgung Hospital (Hospital 2). All patients included in the study exhibited symptoms similar to influenza-like illness and underwent testing for COVID-19, as well as influenza A and B.

The study was conducted between October and February 2022. Upon confirming the presence of influenza A or B, nasal swab samples were collected from eight patients (two males and two females from each hospital). These samples were then sent for sequencing to assess the viral genome. This study was approved by the Ethics Committee of Chinese People’s Liberation Army General Hospital with a waiver of informed consent.

To investigate the clinical characteristics of hospitalized patients with influenza B, we collected data from a total of nine hospitals located in five different provinces across China. These provinces include Hebei, Henan, Beijing, Anhui, and Jiangsu. We extracted various data points from electronic medical records using a standardized data collection form. This included information on demographics, clinical characteristics, laboratory findings, imaging features, bacterial or fungal infections, treatments administered, and outcomes. Additionally, we gathered data from laboratory examinations such as complete blood counts, coagulation profiles, and serum biochemical tests, which encompassed kidney and liver functions, creatine kinase levels, and lactate dehydrogenase levels, among others. Chest CT scans and/or X-ray examinations were performed for all hospitalized patients.

By compiling this comprehensive dataset, we aimed to gain a thorough understanding of the clinical presentation and characteristics of patients admitted to the hospital due to influenza B infection.

### Patients

In this study, patients were eligible for inclusion if they presented at a fever clinic with respiratory symptoms or fever and had received a negative RT-PCR test result for SARS-CoV-2. The study focused on measuring the positivity rates for influenza B between October 2021 and February 2022 in two hospitals located in Beijing. To assess the clinical characteristics of influenza B, data were collected from hospitalized subjects who tested positive for influenza B and negative for COVID-19. These data were obtained from January 7, 2021, to March 9, 2022, and were sourced from nine different hospitals across China, as previously mentioned.

### Data collection

The data for this study were collected through chart reviews of all the subjects who presented at the fever clinic (for the prevalence) or those who were admitted to the hospital due to influenza B infection, using electronic medical records and a standardized data collection form. The data collection process involved three physicians, namely Mingui Lin, Ning Song, and Lihua Xing, who reviewed and recorded the data into the EpiData Software system. To ensure accuracy and consistency, any discrepancies in interpretation were resolved by a fourth physician, Zhantao Zhu, who acted as an adjudicator. To facilitate data management, an online system was developed at www.h6world.cn, where each physician had an account. This system allowed for data entry, review, summary, and organization.

Additionally, data from the China CDC were analyzed for this study. The website used for accessing the China CDC data is https://ivdc.chinacdc.cn/cnic/zyzx/lgzb/.

### Sequencing of the viral genome

To ascertain the viral lineage of influenza B isolated and analyzed using IDseq™ by Vision Medicals.

### Mycoplasma detection

The presence of Mycoplasma infection was detected by the presence of anti-Mycoplasma IgM.

### Detection of secondary infections

Bacterial and fungal infections were detected by the microbial culture of bacterial or fungal pathogens from sputum or broncho-alveolar lavage fluid samples.

### Patients and public involvement

The study obtained consent from patients to acquire respiratory samples for viral diagnosis and to access their medical records. However, beyond providing consent and allowing access to their samples and records, patients did not have a direct role in the study. Publication of the study in open access will provide patients and the general public with access to the findings, allowing them to gain knowledge and insights from the research.

### Statistical analysis

Continuous and categorical variables were presented as Mean ± SD and n (%) respectively. We used the Mann–Whitney U test, χ^2^ test, or Fisher's exact test to compare differences between severe *vs* non-severe and survivor *vs* non-survivor groups as appropriate. A two-sided α less than 0.05 was considered statistically significant. Statistical analyses were done using the SPSS (version 26.0) and GraphPad Prism (version 9.0).

## Results

### Influenza B was a major respiratory pathogen during the flu season in Beijing

**Fig. 1:**
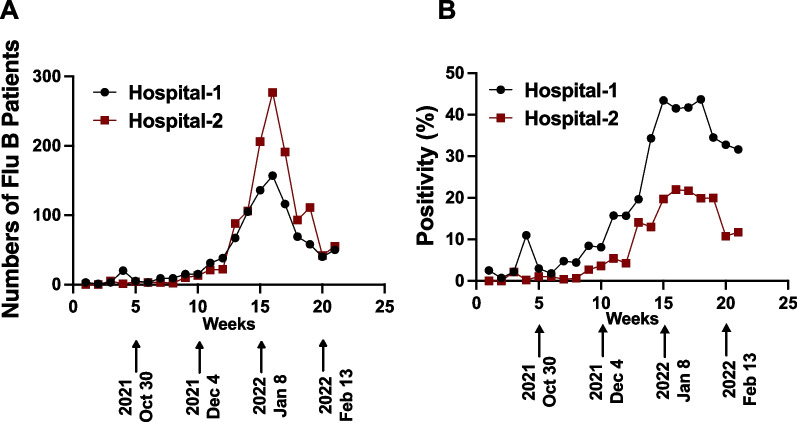
Total number of influenza B positive patients in two hospitals (**A**) and positivity rate for influenza B among those who were tested for influenza-like illness (**B**). No case of influenza A was detected in these hospitals

**Fig. 2:**
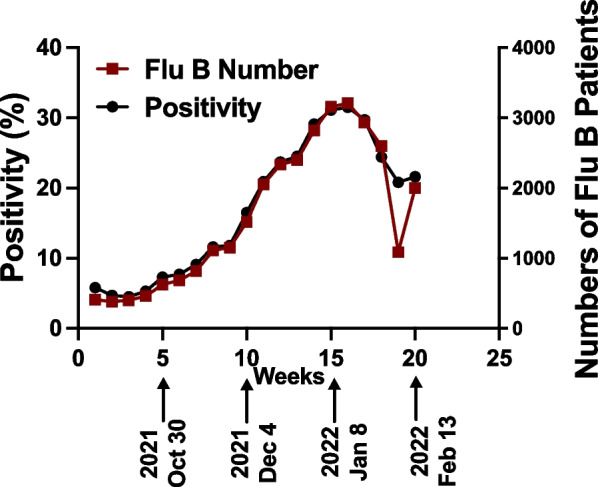
Influenza B activity across China during the influenza season in 2022. Data were analyzed from China CDC along with the positivity rate

In October 2021, there was a significant increase in the number of patients visiting the designated fever clinics in Beijing, and this trend continued until January 2022 (Fig. 1A & B). The COVID-19 testing in all these patients came out to be negative, while the positivity rate for influenza rose from approximately 8% in October 2021 to > 40% in late January 2022. Interestingly, all the cases of influenza were attributed to the influenza B strain, and no cases of influenza A were detected. This observation was consistent with the data from the Centers for Disease Control, China, which also indicated influenza B as the predominant influenza strain during the 2021 flu season (Fig. 2). These findings suggest that in the absence of a widespread COVID-19 during that period in China, influenza B emerged as a prominent respiratory infection.

### Lineage tracing of influenza B

**Fig. 3:**
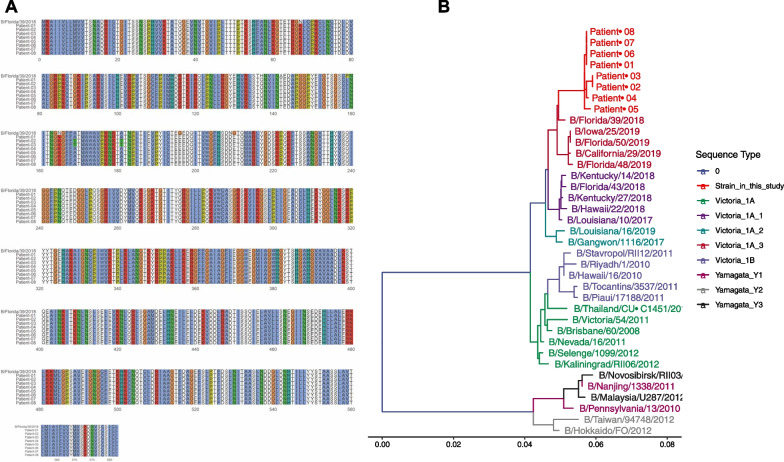
HA genome sequence (**A**) and phylogenetic tree (**B**) of influenza B strain isolated from 8 patients in this study

In order to determine the lineage of influenza B prevalent in Beijing during the 2021 flu season, nasal swab samples from 8 patients (2 males and 2 females from each hospital) were subjected to sequencing to assess the viral genome. The genome sequences of these patients were analyzed, and a phylogenetic tree was constructed to determine the lineage of the influenza B strain. The sequencing results revealed that the prevalent influenza B strain belonged to the Victoria-like lineage. Additionally, the sequencing data showed that the same influenza strains were present in all eight patients, with minor genetic differences compared to the Florida/39/2018 strain (Fig. 3A and B). This information provides insights into the specific lineage and genetic characteristics of the influenza B strain circulating in Beijing during the 2021 flu season.

### Characteristics associated with severe disease in hospitalized patients with influenza B

To understand the key characteristics of disease severity, we analyzed data from 70 patients of which 57 were defined as severe and 13 as non-severe. Severe pneumonia was diagnosed if one of the following major criteria or three or more minor criteria were met. Major criteria: (1) requirement of tracheal intubation for mechanical ventilation; (2) septic shock requiring vasoactive drug therapy even after active fluid resuscitation. Minor criteria: (1) respiratory rate ≥ 30 breaths/min; (2) oxygenation index ≤ 250 mmHg; (3) multiple lobe infiltration; (4) impaired consciousness or disorientation; (5) blood urea nitrogen ≥ 7.14 mmol/L; (6) systolic blood pressure < 90 mmHg requiring fluid resuscitation. Our analysis shows that overall, severe patients were younger in age compared to the non-severe subjects, however, the age difference did not reach statistical significance. Complete blood counts demonstrated no significant differences in total WBC or lymphocyte counts between severe and non-severe groups (Table 2). However, a significant decrease in the platelet counts was observed in the severe group compared to the non-severe group. This was evident as approximately 50% of the subjects in the severe group had thrombocytopenia as defined by platelet count < 150 × 10^9^/L while no patient in the non-severe group had thrombocytopenia (P = 0.008). D-dimer levels, a marker of coagulation, were elevated in the severe group, however, did not reach statistical significance (P = 0.09). Biochemical analysis demonstrated a sharp increase in the markers of liver injury where both ALT and AST were significantly elevated in the severe group compared to the non-severe group. Additionally, an increase was observed in the LDH levels, a marker of cell death and tissue injury in the severe group (*P* = 0.0001). Similarly, an increase was observed in the creatine kinase (*P* = 0.001), indicative of muscle damage. Overall, these results show that a severe disease caused by influenza B is mediated by increased organ injury, thrombocytopenia, and cell death.

**Table 2:** Demographics and clinical characteristics of patients with a severe vs non-severe disease

	Total (n = 70)	Severe (n = 57)	Non-severe (n = 13)	P value
Age (years)	43.2 ± 23.8	40.9 ± 24.1	53.1 ± 20.1	0.127
	Age < 60	29.8 ± 18.3	41.4 ± 15.2	0.11
	Age < 50	23.8 ± 15.0	35.8 ± 13.2	0.07
Sex				
Female	30 (43.0%)	25 (44.0%)	5 (38.0%)	0.77
Male	40 (57.0%)	32 (56.0%)	8 (62.0%)	

Laboratory findings				
	Total (n = 63)	Severe (n = 55)	Non-severe (n = 8)	P value
White blood cell counts (×10^9^ per L)	9.9 ± 7.0	9.39 ± 6.5	13.3 ± 9.8	0.26
Lymphocyte cell counts (×10^9^ per L)	0.8 ± 0.6	0.8 ± 0.6	0.9 ± 0.5	0.14
Plt (×10^9^ per L)	184.5 ± 116.9	176.4 ± 119.4	240.5 ± 83.8	**0.04**
< 100	18 (28.6%)	18 (32.7%)	0 (0)	0.09
< 150	27 (42.9%)	27 (49.1%)	0 (0)	**0.0084**

	Total (n = 59)	Severe (n = 47)	Non-severe (n = 12)	P value
D-Dimmer (μg/ml)	176.8 ± 762.9 (Total)	220.3 ± 851.0	6.45 ± 18.8	0.09

	Total (n = 64)	Severe (n = 53)	Non-severe (n = 11)	P value
Alt (IU/L)	215.7 ± 1158.6	256.7 ± 1273.6	21.8 ± 10.7	**0.01**
Ast (IU/L)	112.9 ± 416.2	131.9 ± 455.8	21.6 ± 8.2	**0.000359**

	Total (n = 59)	Severe (n = 47)	Non-severe (n = 12)	P value
LDH (IU/L)	1234.6 ± 4104.9	1488.6 ± 4573.8	240.1 ± 97.7	0.000179
> 300	35(59.3%)	33(70.2%)	2(16.7%)	0.0019

	Total (n = 56)	Severe (n = 47)	Non-severe (n = 11)	P value
CK (IU/L)	13,427.3 ± 94,803.0	16,626.1 ± 105,500.7	50.7 ± 29.2	**0.001**

Bacterial or fungal infection				
	Total (n = 70)	Severe (n = 57)	Non-severe (n = 13)	P value
Fungal infections	35 (50%)	31 (54.4%)	4 (30.7%)	0.22
* Aspergillus fumigatus*	25 (35.7%)	23 (40.3%)	2 (15.4%)	0.12
Bacterial infections	42 (60.0%)	37 (64.9%)	5 (38.5%)	0.12
* Staphylococcus aureus*	12 (17.1%)	12 (21.1%)	0	0.11
ICU admission	46 (65.7%)	45 (78.9%)	1 (7.7%)	** < 0.0001**
ICU length of stay (days)	6.9 ± 7.7	8.4 ± 7.7	0.85 ± 3.1	**0.000069**
Outcome				
Death	7 (11.4%)	7 (12.3%)	0	0.33

### Characteristics of patients who died of influenza B

In this study, 7 patients succumbed to influenza B infection with age ranges from 1 to 79 years. Patients in the non-survivor groups were younger compared to the survivor group. Similar to severe disease, the levels of LDH and d-dimer were significantly elevated in those who died compared to the survivors. Other markers of the disease severity such as ALT, AST, and CK were elevated in the non-survivor group but did not reach statistical significance (Table 3). Detailed characteristics of these patients are given in Table 4. Interestingly 4 out of 7 subjects were younger than 15 years and all of them were positive for Mycoplasma infection when tested using antigen testing (Table 4). Additionally, 6 out of 7 patients had a secondary infection either by a bacterial or fungal pathogen, suggesting a key role of secondary infections in mortality due to influenza B infections. The only subject that did not have confirmed secondary infection was due to a lack of sample collection given the young age of the patient (1 year).

**Table 3:** Demographics and clinical characteristics of the survivors vs non-survivors

	Total (n = 70)	Non-survivor (n = 7)	Survivor (n = 63)	P value
Age (years)	43.2 ± 23.8	30 ± 30.1	44.7 ± 22.9	0.16
	Age > 65	79	71.7 ± 5.5	0.22
	Age < 60	13.6 ± 12.2	33.7 ± 17.7	0.024
Sex				
Female	30 (42.9%)	2 (37.5%)	28 (51.9%)	0.42
Male	40 (57.1%)	5 (62.5%)	25 (48.1%)	

Laboratory findings				
	Total (n = 63)	Non-survivor (n = 6)	Survivor (n = 57)	P value
White blood cell counts (Í10^9^ per L)	9.9 ± 7.0	9.1 ± 11.6	9.9 ± 6.5	0.35
Lymphocyte cell counts (Í10^9^ per L)	0.8 ± 0.6	0.6 ± 0.2	0.8 ± 0.6	0.37
Plt(Í10^9^ per L)	184.5 ± 116.9	174.1 ± 183.6	185.6 ± 110.1	0.51

	Total (n = 59)	Non-survivor (n = 7)	Survivor (n = 52)	P value
D-Dimmer (μg/ml)	176.8 ± 762.9	430.6 ± 1111.0	142.7 ± 711.8	0.03

	Total (n = 64)	Non-survivor (n = 7)	Survivor (n = 57)	P value
Alt(IU/L)	214.5 ± 1149.4	1577.0 ± 3378.9	47.1 ± 50.4	0.15
Ast(IU/L)	112.1 ± 413.0	522.5 ± 1233.8	62.6 ± 90.1	0.13

	Total (n = 59)	Non-survivor (n = 7)	Survivor (n = 52)	P value
LDH(IU/L)	1234.6 ± 4104.9	7855.5 ± 11,628.8	485.1 ± 400.9	0.02

	Total (n = 57)	Non-survivor (n = 7)	Survivor (n = 50)	P value
CK(IU/L)	13,427.3 ± 94,803.1	124,672.3 ± 289,926.6	339.7 ± 789.4	0.45

Bacterial or fungal infection				
	Total (n = 70)	Non-survivor(n = 7)	Survivor(n = 63)	P value
Fungal infection (Aspergillus, Candida albicans, Cyclospora lucitans, Pneumocystis yersini, cryptococcus)	37 (52.8%)	3 (60.0%)	34 (53.9%)	0.99
Aspergillus infection	26 (37.1%)	2 (28.6%)	24 (38.1%)	0.99
Bacterial infection(S. aureus, Klebsiella pneumoniae, Pseudomonas aeruginosa, Acinetobacter baumannii, Streptococcus pneumoniae, Haemophilus influenzae, Enterococcus faecalis)	38 (54.3%)	5 (71.4%)	33 (52.4%)	0.44
Staphylococcus aureus infection	12 (17.1%)	2 (28.6%)	10 (15.9%)	0.59

**Table 4:** Detailed clinical features of 7 patients who died of influenza B infection

Patient No	Age	Sex	Comorbidities	Chief Complains and symptoms	Hospital duration (days)	Ventilation duration	Secondary infections	Laboratory findings				
								D-Dimmer (μg/ml)	Alt(IU/L)	Ast(IU/L)	LDH(IU/L)	CK(IU/L)
1	60 s	Male	Hypertension, Diabetes,	Dyspnea	9	10	Acinetobacter baumannii,	0.7	19	20	230	46
2	70 s	Female	Interstitial lung disease, Diabetes, Valvular heart disease	Fever, Cough, Sputum, Chill, Dyspnea	17	16	Highly virulent Klebsiella pneumoniae, Pseudomonas aeruginosa, Aspergillus	2950	12	40	Null	38
3	Early adolescence	Male	Mycoplasma infection,	Fever, Cough, Sputum, Dyspnea, Fatigue	2	2	Fungi	35.83	1647	72.15	12,929	716,000
4	Early adolescence	Female	Acute respiratory distress syndrome, Severe pneumonia, DIC, Electrolyte disorders, Hypoproteinemia, Pulmonary hemorrhage, Gastrointestinal hemorrhage, Mycoplasma pneumonia	Fever, Cough, Sputum, Headache, Sore throat, Dyspnea, Fatigue, Hemoptysis	2	2	Staphylococcus aureus	5.9	9120	3320	29,460	29,760
5	Toddler	Male	Hypoalbuminemia, Respiratory failure, ARDS, Mycoplasma pneumoniae infection	Fever, Cough, Sputum, Chill, Headache, Dyspnea, Fatigue	7	7	None	7.3	119	75	3210	57
6	Middle childhood	Male	Mycoplasma infection, Granulocyte deficiency, sepsis, MODS, Coagulation disorders	Fever, Cough, Chill, Headache, Dyspnea, Fatigue, Gasp	4	3	Haemophilus influenzae, Pseudomonas aeruginosa, Aspergillus fumigatus	2.6	32	43	391	-
7	30 s	Female	Septic shock	Fever, Cough, Headache, Sore throat, Runny nose	7	7	Staphylococcus aureus infection	12.05	90	87	913	2133

## Discussion

In this study, we show that influenza B emerged as a leading cause of respiratory infections in the absence of COVID-19 in China during the flu season of 2021–2022. Although our initial data were generated from two fever clinics in Beijing, these trends were true across China as evident by the data obtained from the China CDC Fig. 2 [2]. The emergence of flu B as the major cause of seasonal flu may be contributed to the widespread pandemic controls targeted against COVID-19 that limited the import of viral pathogens from abroad including influenza A. Unlike influenza A, which has multiple hosts including pigs, birds, and other animals [12], humans are the only clinically relevant natural host for influenza B. Other known natural hosts for influenza B are seals [10], however, the transmission of flu B from seals to humans is questionable. It is plausible that, in the absence of influenza A [16], influenza B has re-emerged among the population in China. Further, limited exposure to other viral infections in the previous year due to COVID-19 control measures may have rendered a large fraction of the population susceptible to influenza B infections.

Influenza has been a major threat for centuries including the 1918 flu pandemic that killed a significant proportion of the human population [6]. Even after 1918, there have been many other pandemics caused by influenza viruses. However, these pandemics have been caused by influenza A [7] and so far, no pandemic has been caused by influenza B. However, influenza B causes seasonal flu cases where the disease burden of flu B exceeds that of influenza A every few years. Despite this extensive disease burden caused by flu B, it gained relatively less attention compared to influenza A. Our study shows that despite various control measures, influenza B can infect a significant proportion of patients to cause severe and even lethal diseases.

Our data show that unlike COVID-19, where severe disease and mortality are largely restricted to older subjects [3], influenza B caused severe disease and death even among younger subjects. The average age of hospitalized subjects with severe disease was 41 years while the non-survivors had an average age of 30 years where four out of seven deceased were 15 years or younger. The precise cause of mortality among the younger patients is difficult to decipher given the small sample size, but our data show a major role for secondary infections that were caused by either a bacterial or a fungal pathogen. Interestingly, the four youngest patients who died of influenza B tested positive for Mycoplasma infection. Larger studies are required to understand how a Mycoplasma infection can contribute to the lethality of influenza B infections.

Extensive mortality in younger subjects including the pediatric population has been reported due to influenza B. A Canadian study demonstrated that influenza B had a threefold higher mortality compared to influenza A in children younger than 16 years [14]. Similar reports were observed during the 2010–11 flu season in the USA where 38% of pediatric mortality was attributed to influenza B despite only 26% prevalence. Although the mechanisms by which influenza B contributes to elevated mortality in the pediatric population remain unknown, secondary bacterial infections may be a contributing factor. Mechanistic studies have shown that influenza B is a potent inducer of type I interferon, a cytokine that is already highly expressed in pediatric airways [15]. Of significance, type I interferon is a key factor that renders the host susceptible to secondary bacterial infections [13].

Our study has some limitations such as being a retrospective study. The collection of data retrospectively does not tell us the true prevalence of influenza B infection in the community. Since this study only measured the positivity rate among those presented to the fever clinic, we may have missed patients who did not go to the hospital post symptom onset or those with asymptomatic disease. Further, we observed a 10% mortality rate among the hospitalized subjects, however, the true mortality among the population infected with influenza B remains to be known. Although our study shows that secondary bacterial infections may contribute to lethality, given the small sample size, the precise factors that contribute to the mortality among those infected with influenza B remain to be identified.

## Conclusion

Influenza B with Victoria-like lineage was a major respiratory pathogen during the flu season of 2021–22 in China. The clinical characteristics show evidence of both pulmonary and extrapulmonary manifestations that led to severe disease in infected patients including young subjects. These data show a need for increased monitoring, appropriate vaccination, and early antiviral therapies to limit the morbidity and mortality due to influenza B infections.

## Data Availability

The authors confirm that the data supporting the findings of this study are available within the article, and raw data are available from the corresponding authors, upon reasonable request.

## References

[1] Chen J-M, Chen Y-Q (2022). China can prepare to end its zero-COVID policy. Nat Med.

[2] Centers of Disease Control, China. Summary of Influenza Epidemic Situation in China (as of January 2, 2022) https://ivdc.chinacdc.cn/cnic/zyzx/lgzb/202201/t20220107_255923.htm. Accessed July 19 2022 2022

[3] Elo IT, Luck A, Stokes AC, Hempstead K, Xie W, Preston SH (2022). Evaluation of age patterns of COVID-19 mortality by race and ethnicity from march 2020 to October 2021 in the US. JAMA Netw Open.

[4] Itaya T, Furuse Y, Jindai K (2020). Does COVID-19 infection impact on the trend of seasonal influenza infection? 11 countries and regions, from 2014 to 2020. Int J Infect Dis.

[5] Iuliano AD, Roguski KM, Chang HH, Muscatello DJ, Palekar R, Tempia S, Cohen C, Gran JM, Schanzer D, Cowling BJ (2018). Estimates of global seasonal influenza-associated respiratory mortality: a modelling study. The Lancet.

[6] Johnson NP, Mueller J. Updating the accounts: global mortality of the 1918–1920 "Spanish" influenza pandemic. Bull Hist Med**,** 105–115 (2002). 10.1353/bhm.2002.0022 11875246

[7] Kilbourne ED (2006). Influenza pandemics of the 20th century. Emerg Infect Dis.

[8] Linde A, Rotzén-Östlund M, Zweygberg-Wirgart B, Rubinova S, Brytting M (2009). Does viral interference affect spread of influenza?. Eurosurveillance.

[9] Molinari N-AM, Ortega-Sanchez IR, Messonnier ML, Thompson WW, Wortley PM, Weintraub E, Bridges CB (2007). The annual impact of seasonal influenza in the US: measuring disease burden and costs. Vaccine.

[10] Osterhaus A, Rimmelzwaan G, Martina B, Bestebroer T, Fouchier R (2000). Influenza B virus in seals. Science.

[11] Paget J, Spreeuwenberg P, Charu V, Taylor RJ, Iuliano AD, Bresee J, Simonsen L, Viboud C (2019). Global mortality associated with seasonal influenza epidemics: New burden estimates and predictors from the GLaMOR Project. J Glob Health.

[12] Reperant LA, Kuiken T, Osterhaus AD (2012). Adaptive pathways of zoonotic influenza viruses: from exposure to establishment in humans. Vaccine.

[13] Shahangian A, Chow EK, Tian X, Kang JR, Ghaffari A, Liu SY, Belperio JA, Cheng G, Deng JC (2009). Type I IFNs mediate development of postinfluenza bacterial pneumonia in mice. J Clin Invest.

[14] Tran D, Vaudry W, Moore D, Bettinger JA, Halperin SA, Scheifele DW, Jadvji T, Lee L, Mersereau T (2016). Hospitalization for influenza A versus B. Pediatrics.

[15] Yoshida M, Worlock KB, Huang N, Lindeboom RG, Butler CR, Kumasaka N, Dominguez Conde C, Mamanova L, Bolt L, Richardson L (2022). Local and systemic responses to SARS-CoV-2 infection in children and adults. Nature.

[16] Young G, Peng X, Rebaza A, Bermejo S, De C, Sharma L, Cruz CSD (2020). Rapid decline of seasonal influenza during the outbreak of COVID-19. ERJ Open Res.

